# Influence of River Valleys on Genetic Diversity and Species Distribution Patterns of Cyprididae (Crustacea: Ostracoda) on the Tibetan Plateau

**DOI:** 10.1002/ece3.71759

**Published:** 2025-07-17

**Authors:** Qing Hu, Bingkuan Zhu, Zhihang Ma, Yini Yang, Zhixiong Deng, Shaoqing Wen, Xiaolin Ma

**Affiliations:** ^1^ State Key Laboratory of Estuarine and Coastal Research East China Normal University Shanghai China; ^2^ Institute of Archaeological Science Fudan University Shanghai China; ^3^ Division of Life Sciences and Medicine University of Science and Technology of China Hefei Anhui China; ^4^ School of Public Health, Li Ka Shing Faculty of Medicine The University of Hong Kong Hong Kong SAR China; ^5^ MOE Key Laboratory for Biodiversity Science and Ecological Engineering, School of Life Science Fudan University Shanghai China

**Keywords:** geographical isolation, mitonuclear discordance, phylogenetic analysis, river valleys, species composition

## Abstract

River valleys are recognized as significant ecological barriers that impact gene flow between species adapted to distinct habitat types. The Tibetan Plateau, with its diverse habitats intersected by numerous river valleys, serves as a focal point for biodiversity research. Although previous studies have focused on plants and terrestrial animals, research on the genetic diversity of aquatic species influenced by river valleys in the Tibetan Plateau is limited. In this study, we utilized mitochondrial and nuclear genetic markers to examine variations in species composition and biodiversity among 18 Cyprididae communities (106 individuals) on the Tibetan Plateau, separated by three distinct river valleys: the Zhajia Zangbu River, the Nujiang River, and the Yarlung Zangbo River. Our phylogenetic analysis based on COI sequences revealed that the sampled communities are clustered into three genetic branches, which correspond to nine clades including two cryptic lineages. Among the 15 communities analyzed, eight exhibited mitochondrial‐nuclear discordance, indicating complex evolutionary dynamics such as hybridization or incomplete lineage sorting. Furthermore, significant differences in species composition and genetic diversity were observed among the three river valleys, potentially influenced by altitude and other environmental factors. This study offers new insights into the genetic diversity and species distribution of Cyprididae on the Tibetan Plateau, highlighting the role of geographical isolation induced by river valleys in shaping regional endemism and contributing to a broader understanding of biogeographical barriers in aquatic species.

## Graphical abstract

The Tibetan Plateau, characterized by its rich biodiversity and numerous intersecting river valleys, serves as a prime location for biodiversity research. In this study, we employed mitochondrial and nuclear genetic markers to investigate variations in species composition and biodiversity among 18 populations of Cyprididae (comprising 106 individuals) across the Tibetan Plateau, specifically separated by three major river valleys: the Zhajia Zangbu River, the Nujiang River, and the Yarlung Zangbo River.

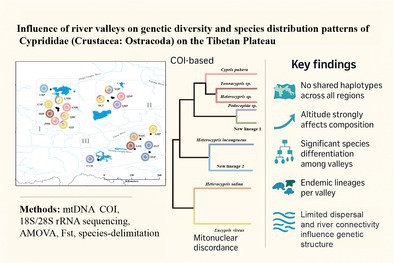

## 1 Introduction

River valleys have long been recognized as significant ecological barriers that restrict gene flow between species with distinct habitat requirements (Playford et al. 1993; Li et al. 2009; Hazzi et al. 2018; Yang et al. 2022). Rivers, especially those with swift currents, act as physical barriers that impede the movement of species with limited dispersal capabilities (Davis et al. 2018). This hindrance to gene flow ultimately leads to genetic differentiation and can even result in the emergence of distinct species or sub‐species (Oliveira et al. 2018). For example, Maldonado‐Coelho et al. (2013) found that the Tocantins River historically served as a barrier, causing population divergence among fire‐eye antbirds in the Amazon. Similarly, the Changhua River and its tributaries isolated populations within the mountainous regions of Hainan Island, leading to significant genetic differentiation of Metapetrocosmea peltate (Li et al. 2020). Moreover, vegetation showed geographical isolation between the Dadu River valley and the Yalong River valley in the plateau area (Han et al. 2016). Many valleys within the Tibetan Plateau have also been identified as biogeographical barriers for numerous species (Jin and Ou 2000; Qiao et al. 2020). The Tibetan Plateau is intersected by numerous river valleys (Yang et al. 1982), providing diverse habitats and serving as a focal point for biodiversity research (Bai‐ping et al. 2002). Although previous studies have primarily focused on plants and terrestrial animals (Han et al. 2016; Qiao et al. 2020), research on the genetic diversity of aquatic organisms influenced by river valleys in the Tibetan Plateau remains limited.

Aquatic organisms, such as ostracods, are small crustaceans protected by a calcified, dorsally hinged carapace that resembles the structure of bivalve shells (Klaoudatos and Klaoudatos 2008), as model systems for paleoenvironmental, paleoclimate, environmental, and evolutionary studies (e.g., Ghafor and Najaflo 2022; Sinha and Singh 2021). They inhabit a wide range of environments, from freshwater to marine environments, particularly within benthic and periphyton communities (Martens et al. 2008). Approximately 2300 species of living nonmarine ostracods have been described, representing the majority of Ostracoda (Meisch, Scharf, et al. 2019; Meisch, Smith, and Martens 2019; Almeida et al. 2021). Within this diverse taxonomic unit, the family Cyprididae Baird, 1845 is widely recognized as one of the most taxonomically and ecologically prominent lineages within the Podocopida order (Martens et al. 2008), exhibiting remarkable ecological adaptability to freshwater habitats including wide distribution, regional endemism, environmental sensitivity, and high genetic diversity (Martens and Rossetti 1997; Smith et al. 2015). Species within the family Cyprididae, such as *Cypris pubera* Müller, 1776, typically inhabit lake margins (littoral zones) or occasionally slightly saline water bodies prone to drying out (Altınsaçlı and Griffiths 2002). Additionally, cosmopolitan species like *Eucypris virensis* Jurine, 1820, and *Heterocypris incongruens* Ramdohr, 1808 are commonly found in temporary habitats (Meisch 2000). Despite having some degree of swimming ability, Cyprididae exhibit limited mobility due to the high energy expenditure associated with movement (Rahman et al. 2022), hindering effective gene flow with distant populations and thereby maintaining genetic differences (Martens et al. 2008). Consequently, the family Cyprididae demonstrates a high level of endemicity, with only approximately 10% of the described species distributed across more than one zoogeographical province (Meisch et al. 2024).

Due to regional endemism and high genetic diversity, the taxonomy of Cyprididae poses significant challenges (Meisch 2000). Although morphological identification serves as the universal basis of taxonomic classification across all taxonomic units, the family Cyprididae is characterized by subtle intra‐species morphological variations and cryptic species complexes that complicate this process, often necessitating extensive expertise and meticulous microscopic examination for accurate identification (Smith 2000; Sánchez‐González et al. 2004; Karan‐Žnidaršič et al. 2018). However, relying solely on morphological identification is not only time‐consuming but also subject to the taxonomic expertise of the classifier (De Deckker 1978; Serrana et al. 2019). Additionally, some studies have focused on genera and species within the family Cyprididae based on molecular markers. For example, a study on the halophilic nonmarine ostracod using molecular markers, such as mitochondrial COI and nuclear ribosomal DNA (28S), revealed multiple cryptic species within this morphologically conserved group (Kilikowska et al. 2024). Similarly, Kong et al. (2014) clarified the phylogenetic position of the genus Chrissia within the subfamily Herpetocypridinae and the family Cyprididae based on 18S rRNA. Moreover, the phylogenetic relationships within Cyprididae were explored by utilizing the 18S rRNA gene and a portion of the 28S rRNA gene (Hiruta et al. 2016). Despite numerous studies, the genetic relationships of families within Cyprididae remain controversial (Danielopol and Mckenzie 1978; Maddocks 1992; Martens 1998; Meisch 2000; Liebau 2005; Martens et al. 2008; Karanovic 2012). In China, extensive research has been conducted on ostracods from sedimentary stages in Earth's geological record (Li et al. 2010; Wang et al. 2017; Ye et al. 2022). However, studies on extant Cyprididae are lacking, with a primary focus on documenting new species (Ma and Yu 2020).

In this study, we utilized mitochondrial and nuclear genetic markers to elucidate the differences in species composition and biodiversity among 18 Cyprididae communities on the Tibetan Plateau. These communities are distributed across three distinct river valleys: the Zhajia Zangbu River (RVI), the Nujiang River (RVII), and the Yarlung Zangbo River (RVIII). Additionally, we explored potential factors influencing these differences in species composition. Our results significantly enhance the understanding of genetic diversity and regional endemism among ostracod species on the Tibetan Plateau.

## 2 Materials and Methods

### 2.1 Study Valleys and Sampling

**FIGURE 1:**
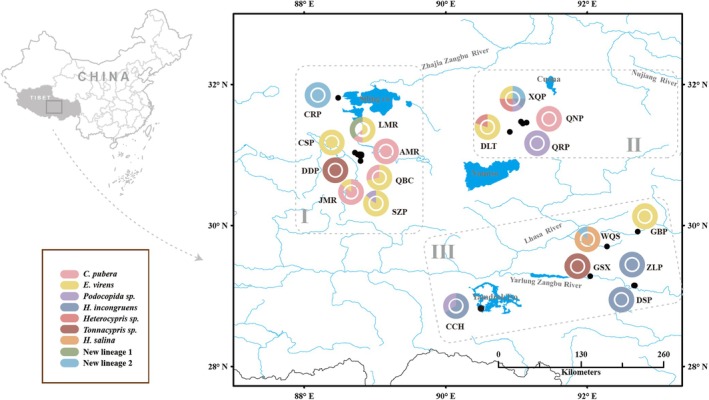
Geographic distribution of sampling locations on the Tibetan Plateau. Color codes indicate the geographical locations of Ostracoda species. For lake abbreviations, see Table 1.

From 2012 to 2019, Ostracoda specimens were collected from 18 out of 153 water‐bodies on the Tibetan Plateau (Figure 1 and Table 1), utilizing a plankton net (mesh size: 125 μm) hauled at three different sites per locality. Subsequently, samples gathered from the same water‐body were combined and preserved in 95% ethanol for further analyses. The sampling regions were primarily categorized into three river valleys corresponding to Zhajia Zangbu River (abbreviated as “RVI”), Nujiang River (RVII) and Yarlung Zangbo River (RVIII) based on the flow direction and distribution of rivers within the sampling area. Particularly, RVI originates from the Tanggula Mountains, whereas the eastern section of Nyenchen Tanggula Mountains serves as the watershed between RVII and RVIII. The diversity in mountain ranges, topography, altitude, climate, and other factors has led to variations in the surrounding environments of the three river valleys (Guan and Chen 1980). All locations where Ostracoda were collected were characterized by small and shallow water‐bodies, with an average altitude of 4400 m above sea level (ranging from 3398 to 4942 m). The average altitudes of the three river valleys correspond to 4798, 4597, and 3756 m, respectively. The maximum geographical distance and elevation difference between the sampling sites are approximately 498 km and 1544 m, respectively.

**TABLE 1:** List of localities in which Ostracoda was detected (name, abbreviation and geographical position) on the Tibetan plateau and genetic characteristics of sequenced individuals.

Locality (abbreviation)	Latitude, longtitude	Altitude (m)	*N*_1_	Mitochondrial marker (COI)				Nuclear marker (18S + 28S)		
				*N*_2_	*N*_3_	Species^a^	Haplotypes	*N*_4_	*N*_5_	Species^b^
Zhajia Zangbu River (I)										
Angmuruo (AMR)	31.00 N, 88.76 E	4912	8	8	2	*C. pubera*	AMRa, cp_hap1	3	8	*Stenocypris* sp.
Ciru (CRP)	31.79 N, 88.45 E	4511	8	8	1	*Heterocypris* spp.	CRPa	8	8	Undefined
Chasuo (CSP)	31.07 N, 88.69 E	4770	8	8	1	*E. virens*	ev_hap1	7	8	Undefined
Dadong (DDP)	30.99 N, 88.76 E	4940	7	7	2	*Tonnacypris* spp.	DDPa, t_hap1	5	7	Undefined
Jiangmuru (JMR)	31.07 N, 88.69 E	4767	7	7	2	*E. pigra* , *E. virens*	ep_hap1, JMRb	7	7	*Stenocypris* sp. undefined
Langmare (LMR)	31.06 N, 88.69 E	4776	8	8	5	*E. virens* , *E. pigra Podocopida* spp.	ev_hap1, ev_hap2, LMRa, LMRb, LMRc	5	8	*Stenocypris* sp.
Qubucun (QBC)	31.07 N, 88.69 E	4767	7	7	3	*E. virens* , *E. pigra*	ev_hap1, ep_hap1, ev_hap2	6	7	Undefined
Shenzha (SZP)	30.99 N, 88.76 E	4942	8	8	2	*E. virens* , *Podocopida* spp.	ev_hap1, p_hap1	6	8	Undefined
Nujiang River (II)										
Dalietang (DLT)	31.40 N, 90.87 E	4533	5	5	2	*E. virens* , *H. salina*	ev_hap1, hs_hap1	1	5	Undefined
Quenong (QNP)	31.49 N, 91.14 E	4635	6	6	1	*C. pubera*	cp_hap1	2	6	*Stenocypris* sp.
Qiri (QRP)	31.49 N, 91.14 E	4639	3	3	1	*Podocopida* spp.	p_hap1	1	3	Undefined
Xianqiong (XQP)	31.50 N, 91.08 E	4579	8	8	5	*Heterocypris* spp., *Podocopida* spp., *H. incongruens* , *H. salina* , *E. virens*	XQPa, p_hap1, XQPb, hs_hap1, XQPc, XQPd	7	7	*Cyprididae gen*. sp., *Ilyocypris* sp.
Yarlung Zangbo River (III)										
Caicuohu (CCH)	28.78 N, 90.45 E	4425	3	3	3	*H. incongruens* , *Podocopida* spp.	CCHa, CCHb, CCHc	3	3	*H. incongruens* , undefined
Dingsang (DSP)	29.18 N, 92.67 E	3400	2	2	1	*H. incongruens*	DSPa	2	2	*H. incongruens*
Gongbujiangda (GBP)	29.91 N, 93.15 E	3459	6	6	1	*E. virens*	GBPa	n.a.	n.a.	n.a.
Gongsangxian (GSX)	29.29 N, 92.06 E	3513	1	1	1	*Tonnacypris* spp.	t_hap1	n.a.	3	n.a.
Wenquanshanzhuang (WQS)	29.70 N, 92.24 E	4341	7	7	1	*H. salina* , *Heterocypris* spp.	WQSa	5	5	*Heterocypris salina* , undefined
Zhenglang (ZLP)	29.18 N, 92.67 E	3398	4	4	1	*H. incongruens*	ZLPa	2	4	*H. vandouwei*

*Note:* *N*_1_: number of individuals used for genetic analyses; *N*_2_: number of individuals for COI sequencing; *N*_3_: haplotypes for COI sequencing; *N*_4_: number of individuals for 18S sequencing; *N*_5_: number of individuals for 28S sequencing.

^a^ Species identified by COI.

^b^ Species identified by 18S + 28S.

### 2.2 DNA Extraction and Sequencing

An average of six Ostracoda specimens per locality were randomly selected and placed individually at the bottom of 0.2 mL tubes for subsequent molecular analyses (Table 1). Prior to DNA extraction, the Ostracoda shells were mashed using a sterile gun tip. DNA was then extracted from individual specimens in a total volume of 50 μL containing proteinase K (10 mg/mL; MERCK, Germany) and H3 buffer (10 mM Tris–HCl, 0.05 M KCl, 0.005% Tween 20, 0.005% NP‐40). Specimens were incubated overnight at 55°C in a water bath with mild shaking. After heat inactivation of the proteinase K for 12 min at 95°C, the tubes were cooled to 4°C, briefly centrifuged, and then stored at −20°C until further use.

The 680‐bp fragment of the mitochondrial cytochrome c oxidase subunit I (COI) gene was amplified using the standard primers LCO1490 and HCO2198, as described by Folmer et al. (1994). The PCR reaction mixture, with a total volume of 25 μL, contained 1 μL of genomic DNA, 1 μL of each primer at a concentration of 10 μM, 9.5 μL of ddH_2_O, and 12.5 μL of 2 × Hieff PCR Master Mix (with dye). The cycling conditions for the PCR were as follows: an initial denaturation at 94°C for 1 min, followed by 40 cycles of denaturation at 94°C for 30 s, annealing at 55°C for 30 s, and extension at 72°C for 40 s. Finally, a 10‐min extension step was performed at 72°C. Two nuclear gene fragments (a 1900‐bp fragment of 18S rRNA gene and a 300‐bp fragment of 28S rRNA gene) were amplified using the primers EuK‐63F/Eukarya‐1818R (Lepere et al. 2011) and 28S rRNA‐F/28S rRNA‐R (Nunn et al. 1996). The amplification cycling conditions for 18S and 28S were as follows: incubation at 95°C for 3 min, then 35 cycles of 3 min at 95°C, 50 s at 55°C for 28S and 57°C for 18S, and 1 min at 72°C; this was followed by a final incubation for 5 min at 72°C. To identify and exclude multiple heterozygous sequences of 18S and 28S, cloning was performed with the protocol used in the previous research (Ni et al. 2019; Wang et al. 2021). Ten clones of each PCR product were sequenced. All COI, 18S, and 28S PCR products were sequenced using forward or/and reversed primers on an ABI PRISM 3730 DNA capillary sequencer by Sangon Biotech Co. Ltd. (Shanghai, China). For the clones of nuclear genes, only identical sequences occurring more than twice were retained for the further analysis. Then, sequence chromatograms were thoroughly examined, and any scoring errors were manually corrected using MEGA X (Kumar et al. 2018). All new sequences have been submitted to GenBank under accession numbers: COI: PP999775‐PP999879, 28S: PQ000132‐PQ000237, and 18S: PQ012688‐PQ012754.

### 2.3 Sequence Alignment and Phylogenetic Analyses

The mitochondrial COI gene sequences were aligned using the Clustal W algorithm (Thompson et al. 1994). DnaSP 6 (Rozas et al. 2017) was utilized to identify unique haplotypes. Subsequently, each unique haplotype was aligned with 37 reference sequences obtained from GenBank (Table S3) using the Clustal W algorithm in MEGA X. *Macroscapha opaca* was selected as an outgroup (Brandao et al. 2010). For the two nuclear gene fragments (18S and 28S), unique alleles were identified in DnaSP 6, and then aligned with 27 (18S) and 25 (28S) reference sequences from GenBank (Table S4). *Macrocypris* sp. was chosen as an outgroup for 18S and 28S (Hiruta et al. 2016), respectively. Subsequently, a concatenated alignment of 18S and 28S genes (referred to as “18S + 28S”) was created including reference sequences available for both 18S and 28S.

A Bayesian phylogenetic tree for the COI gene was constructed using BEAST 2 (Bouckaert et al. 2014). Trees were recorded every 1000 generations over a total of 10,000,000 generations. After discarding the first 25% as burn‐in, the remaining 10,000 trees were summarized using TreeAnnotator. The best substitution model was determined based on the corrected Bayesian Information Criterion in IQtree (Minh et al. 2020). Tracer v 1.6 (Rambaut et al. 2018) was used to verify that enough generations were computed. Similarly, a Bayesian phylogenetic tree for the concatenated 18S + 28S dataset was constructed in BEAST 2 using similar parameters.

### 2.4 Detection of Mitochondrial Lineages and Phylogenetic Analyses

Three independent species‐delimitation methods were applied to delineate clades within the phylogenetic tree for COI (Deng et al. 2022). These methods included Automatic Barcode Gap Discovery (ABGD) (Puillandre et al. 2012), the Bayesian implementation of Poisson Tree Processes (bPTP) (Zhang et al. 2013), and the General Mixed Yule Coalescent (GMYC) model (Pons et al. 2006). The ABGD method was applied through the online tool (http://wwwabi.snv.jussieu.fr/public/abgd/) with default settings to categorize sequences into hypothetical species based on the barcode gap. For the bPTP analysis, we utilized the bPTP webserver (http://species.h‐its.org/ptp/) with the following parameters: 100,000 MCMC generations, a thinning interval of 100, and a 25% burn‐in period. GMYC modeling was conducted using the “splits” package in R 3.6.1 (R Development Core Team 2013). The input phylogenetic tree for these analyses was generated using BEAST 2.

To assess intraspecific genetic variation and community relationships, haplotype networks of three commonly reported species (i.e., *C. pubera* , *E. virens* , and *H. incongruens* ) based on COI were constructed using HAPLOVIEWER (Salzburger et al. 2011). The *C. pubera* network included haplotypes from Canada, China, India, and Turkey (Table S5). The *E. virens* network had haplotypes from Australia, China, and Europe (Table S6). The *H. incongruens* network comprised haplotypes representing four regions (Belgium, China, India, and Turkey; Table S7).

### 2.5 Species Composition, Diversity and Genetic Variation Assessment Based on COI

To compare species composition (proportion) and species diversity (H′ Shannon‐Wiener index) among three river valleys, Pearson's Chi‐squared test and *t* test were performed using R (Berry et al. 2021), respectively. Statistical significance was determined at *p* < 0.05. To analyze the genetic variance, the genetic variance was partitioned into three levels: (1) among different regions, (2) among communities within each region, and (3) within individual communities. Then, a hierarchical analysis of molecular variance (AMOVA) was conducted using Arlequin 3.5.2 (Excoffier et al. 2005). To estimate genetic differentiation among Cyprididae communities, pairwise *F*_ST_ values were calculated for 18 communities in Arlequin 3.5.2, following the method of Weir and Cockerham (1984). Furthermore, the influence of geographical variables (geographical distance, longitude, latitude and altitude) on genetic variation was evaluated through principal components analysis (PCA) and correlation analysis between these variables and pairwise *F*_ST_ values using R.

## 3 Results

### 3.1 Genetic Diversity in Family Cyprididae Communities

A total of 106 individuals from the Ostracoda taxonomic unit (specimens belonging to the family Cyprididae) were successfully sequenced at the COI locus, resulting in a dataset of 526 bp (Table 1). Twenty‐five unique haplotypes were identified, with no haplotypes shared across all three regions (Figure 1). Three haplotypes (ev_hap1, cp_hap1, and p_hap1) were shared between regions RVI and RVII. One haplotype (t_hap1) was shared between RVI and RVIII, whereas no haplotypes were shared between RVII and RVIII. The most frequently observed haplotype, ev_hap1, was widely distributed in regions RVI and RVII and was shared by individuals from five communities. Furthermore, nine out of the 18 communities harbored only a single haplotype, whereas four communities exhibited three or more haplotypes. For the two nuclear loci, 70 and 99 sequences were obtained at the 18S and 28S loci, resulting in 24 and 23 unique alleles. All alleles were found to be homozygous (Table S1). Among these alleles, 18S_A and 28S_A were the most prevalent alleles in individuals, predominantly restricted to regions RVI and RVII and absent in RVIII.

### 3.2 Phylogenetic Analysis of COI Sequences

**FIGURE 2:**
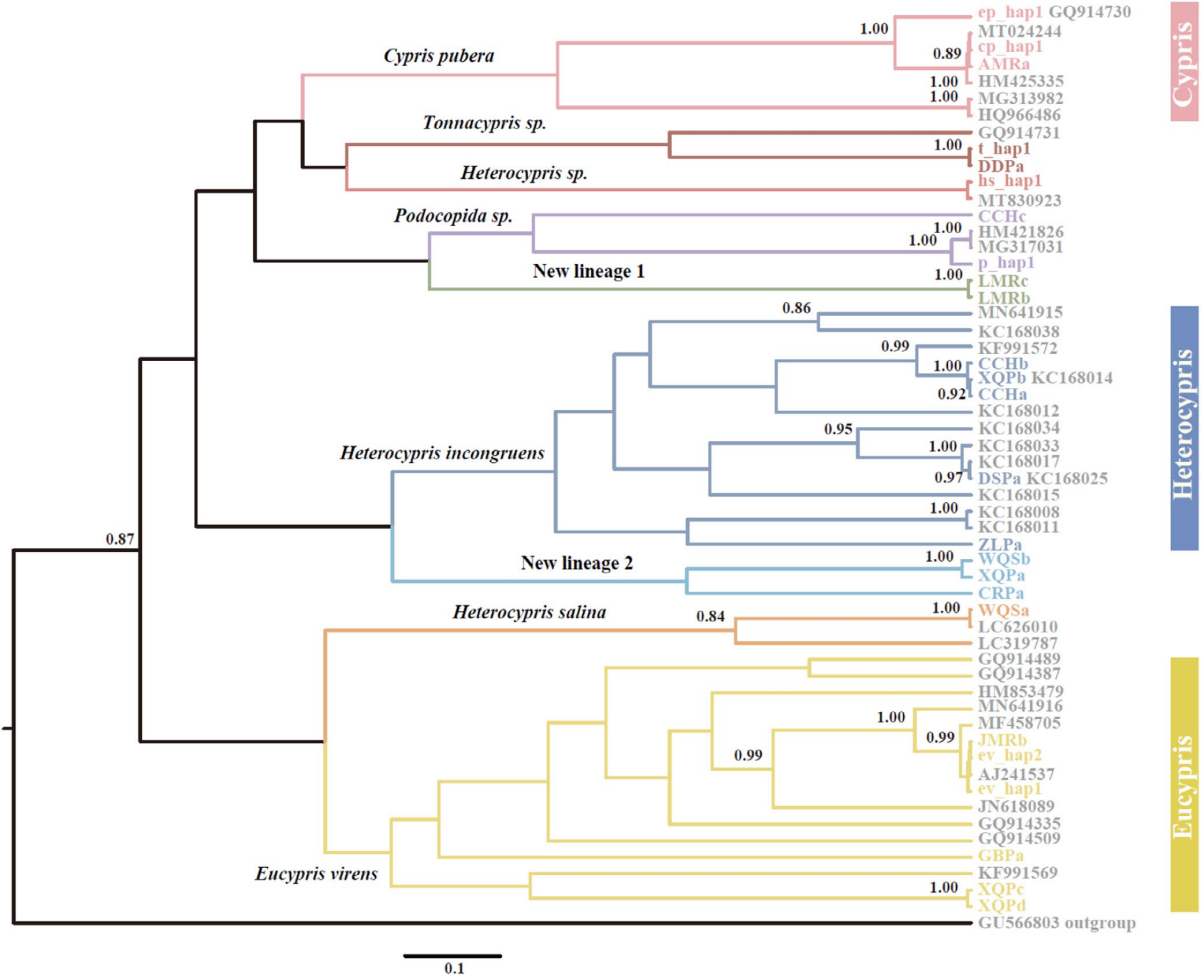
Bayesian phylogenetic tree and species delimitation results for Ostracoda from the Tibetan Plateau based on the COI gene (527 bp). Only posterior probabilities > 0.70 are shown. Species delimitation according to the GMYC and bPTP methods is indicated. For the bPTP method, the Bayesian support value for clade membership is shown. *Macroscapha opaca* was used as an outgroup. Codes of Ostracoda haplotypes from the Tibetan Plateau are provided in Table 1.

Phylogenetic analysis of COI sequences revealed the genetic diversity within the family Cyprididae specimens sampled from 18 waterbodies across the Tibetan Plateau. Three major genetic branches were identified, consisting of nine distinct clades: *Cypris pubera* , *Tonnacypris* sp., *Heterocypris* sp., *Podocopida* sp., *Heterocypris incongruens* , *Heterocypris salina* , *Eucypris virens* , and two new lineages (referred to as new lineage 1 and new lineage 2; see Figure 2). Interestingly, two cryptic lineages were discovered, with new lineage 1 exclusively found in LMR waterbodies within RVI, whereas new lineage II, consisting of three haplotypes (CRPa, XQPa, and WQSb), was distributed across all three valleys. A clear geographical pattern emerged among the nine lineages: some were widely distributed in three regions ( *E. virens* , *Podocopida* sp., and new lineage 1), some were found in only two regions ( *C. pubera* shared by RVI and RVII, *H. incongruens* shared by RVII and RVIII, and *Tonnacypris* sp. shared by RVI and RVIII), and others were endemic to a single region (new lineage 2 in RVI, *Heterocypris* sp. in RVII, and *H. salina* in RVIII).

### 3.3 mtDNA Haplotype Networks of *C. pubera* , *E. pigra* , and *H. incongruens*

The mtDNA haplotype networks for *C. pubera* , *E. virens* , and *H. incongruens* were analyzed in this study. For *C. pubera* , three Chinese haplotypes were identified along with two haplotypes from Canada (Figure S1). Interestingly, one haplotype was found to be shared among China, India, and Turkey. The *E. virens* network included five Chinese haplotypes, with one haplotype shared by China, Australia, and Europe (Figure S2). In the case of *H. incongruens* , five Chinese haplotypes were identified, along with 25 haplotypes from Belgium, India, and Turkey (Figure S3). Notably, one haplotype, located at the center of the star‐like network, was shared by China and Belgium.

### 3.4 Nuclear Loci Phylogenetic Analysis

**FIGURE 3:**
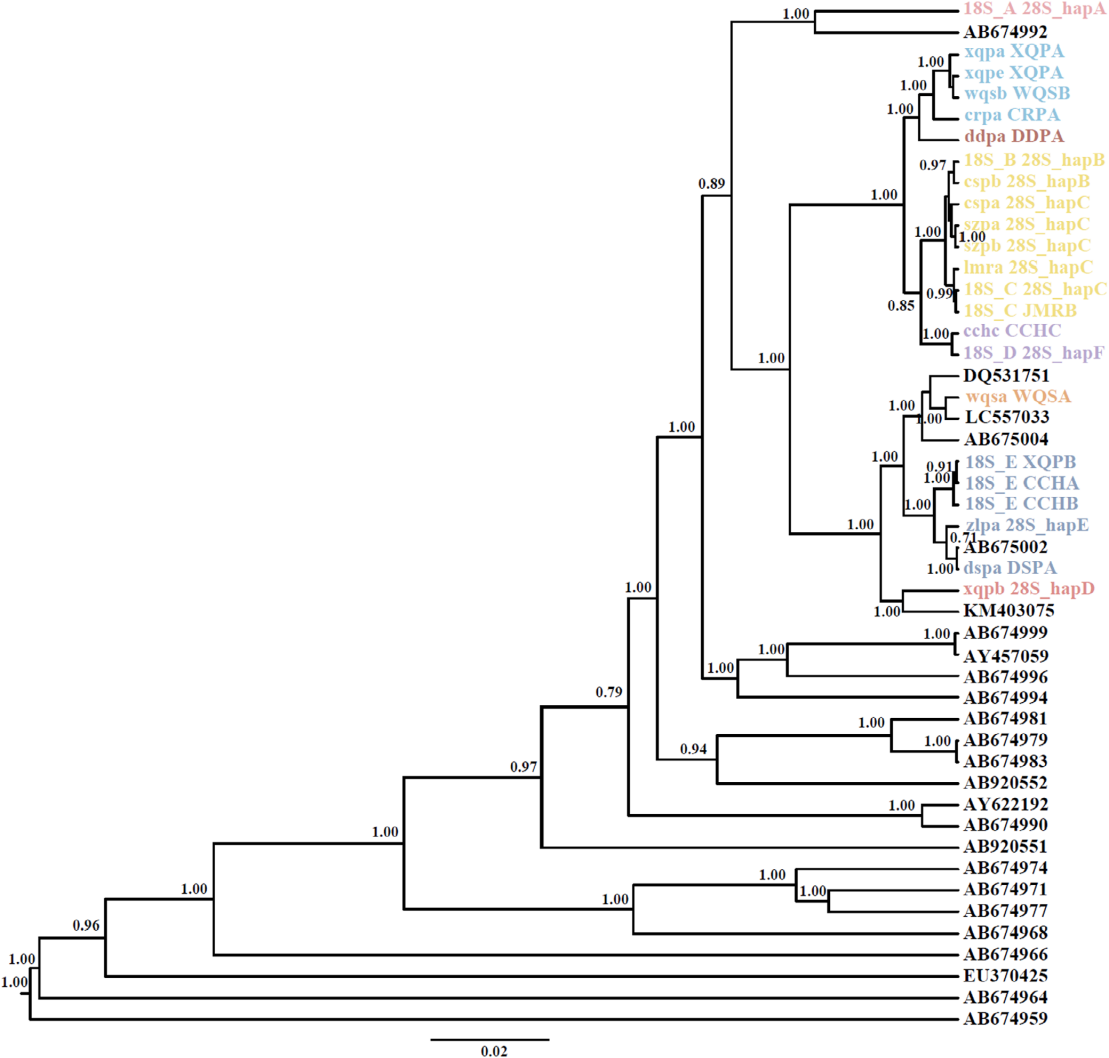
The Bayesian phylogenetic tree of 18S + 28S (2131 bp) of the family Cyprididae (Crustacea: Ostracoda) from the Tibetan Plateau. Only posterior probabilities > 0.70 are shown.

Our analysis of nuclear loci 18S + 28S revealed the presence of eight distinct clades in the phylogenetic tree (Figure 3). However, only four clades could be confidently defined due to a lack of reference sequences, which corresponded to *Stenocypria* sp., *H. salina* , *H. incongruens* , and *Cyprididae* gen. sp. Mitochondrial‐nuclear discordance was observed in eight out of 15 communities (excluding three communities with insufficient data; see Figure S4). Among these communities, four exhibited discordance solely at the 18S locus, whereas four showed discordance at both the 18S and 28S loci (Figure S5). Interestingly, one particular nuclear 18S + 28S clade was found to encompass multiple COI haplotypes, such as 18S_A 28S_hapA, which contained three distinct COI haplotypes, and ddpa DDPA, which contained two different COI haplotypes.

### 3.5 Genetic Differentiation and Geographical Correlation Analysis

**FIGURE 4:**
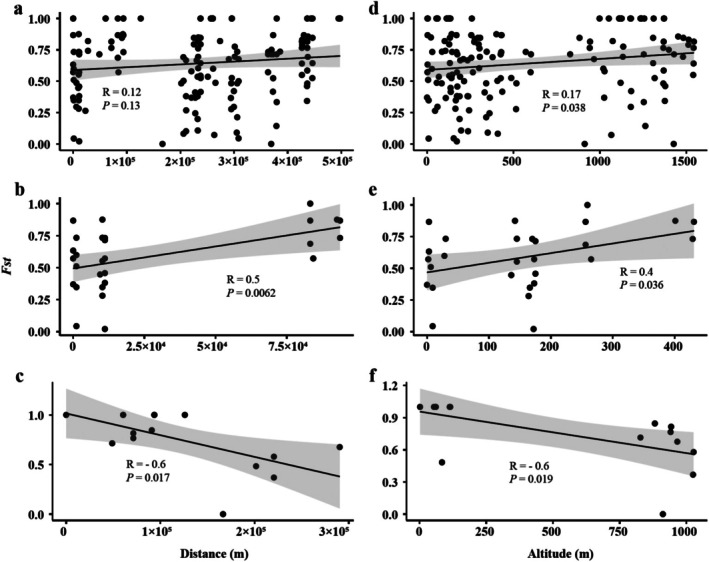
Correlation analysis between genetic differentiation (*F*_ST_) and geographical variables (distance and altitude) across different regions. (a–c) *F*_ST_ vs. geographical distance across all regions, RVI and RVIII, respectively. (d–f) *F*_ST_ vs. geographical altitude across all regions, RVI and RVIII, respectively.

Based on the analysis of COI, the Molecular Variance (AMOVA) results indicated that the majority of variation was observed among communities within regions (70.79%; refer to Table 2). Pairwise *F*_ST_ values among Cyprididae communities ranged from 0 to 1 (Table S2), representing the level of genetic differentiation between communities, with higher values indicating greater differentiation. It is worth noting that there was no significant correlation between pairwise *F*_ST_ values and geographical distance (*p* = 0.133; see Figure 4a). In terms of the correlation between *F*_ST_ values and geographical distance within regions, a significant linear correlation was observed in RVI and RVIII (*p* < 0.05; refer to Figure 4b,c), respectively. However, no significant correlation was found in RVII (*p* = 0.98; not shown in Figure 4 due to limited sampling points). When Principal Component Analysis (PCA) was applied to geographical predictors, the first and second components explained 75.6% and 19.2% of the variability, respectively (Figure S6). The first component was closely associated with altitude. Additionally, a significant linear correlation between *F*_ST_ values and altitude was observed in all regions, RVI, and RVIII (*p* < 0.05; see Figure 4d–f).

**TABLE 2:** Hierarchical AMOVA for family Cyprididae based on COI.

Source of variation	df	Explained variation (%)	*p*
Among regions	2	0.86	< 0.001
Between communities within regions	15	70.79	< 0.001
Within communities	87	28.34	0.340

*Note:* Among regions (i.e., Zhajia Zangbu River valley, Nujiang River valley and Yarlung Zangbo River valley) variation is estimated in relation to within‐region and within‐communities components.

### 3.6 Species Composition Variation Among River Valleys

**FIGURE 5:**
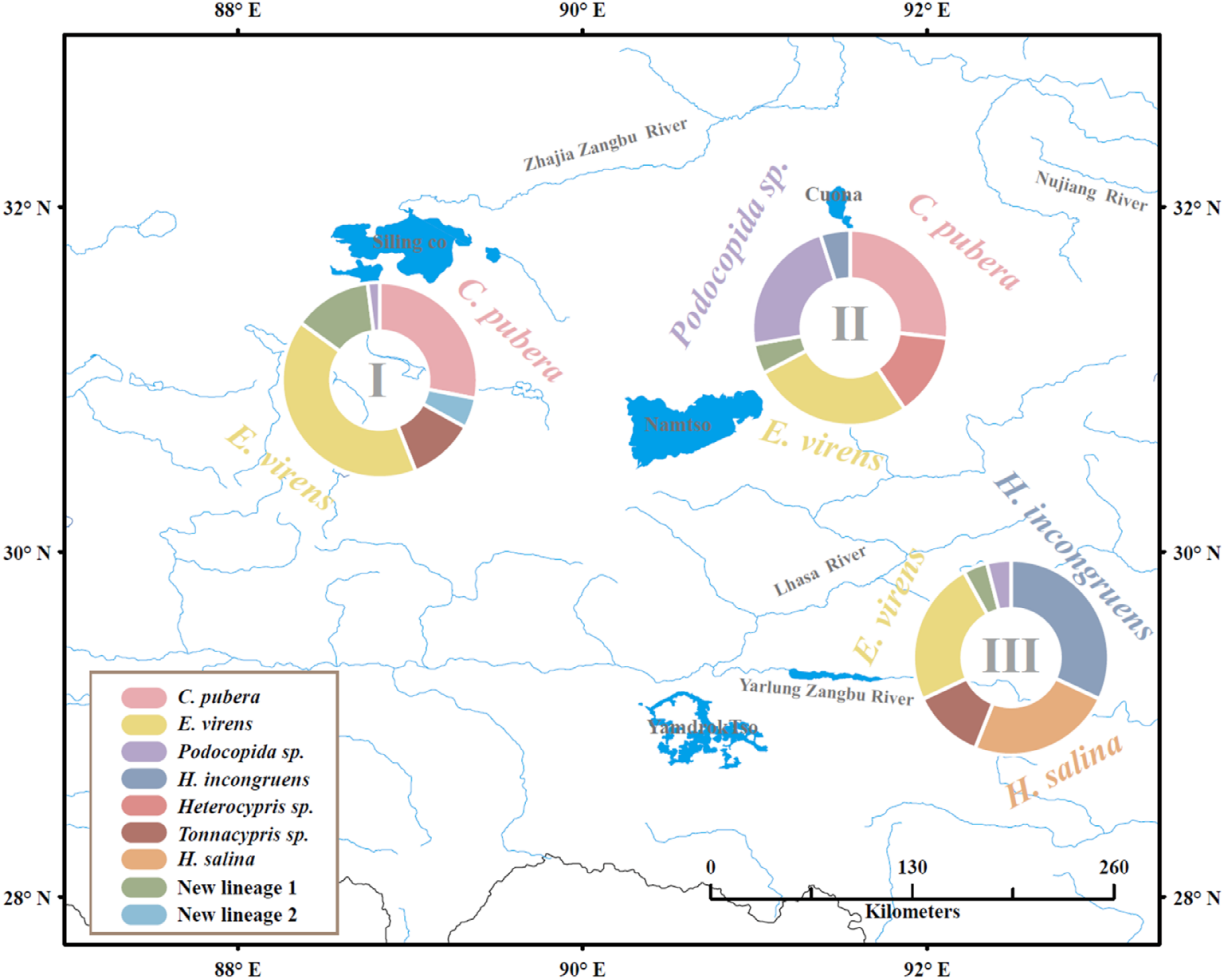
Donut chart depicting species composition in three different river valleys around Namtso Lake based on COI. The regions are labeled as I, II, and III. Each color represents a different species, as indicated in the legend.

Species composition varied significantly among the three river valleys (*χ* ^2^ = 81.26, df = 16, *p* < 0.001), indicating a substantial difference in species distribution. At RVI, *E. virens* was the most abundant species, followed by *C. pubera* , whereas *Podocopida* sp. was scarce (Figure 5). In RVII, *E. virens* and *C. pubera* were the most abundant, with *Podocopida* sp. following. Conversely, at RVIII, *H. incongruens* was the dominant species, followed by *H. salina* and *E. virens* . Despite these differences, species diversity did not vary significantly across the three valleys (*F* = 0.125, df = 2, *p* = 0.883), with slightly lower diversity observed at RVI. Three species were present in all three river valleys (e.g., *E. virens* , *Heterocypris* sp. and new lineage I), but their proportions differed. Each valley also had its own endemic species including new lineage II, *Heterocypris* sp., and *H. salina* .

## 4 Discussion

### 4.1 Diversity and Biogeography: Insights From Three Genera

The majority of species identified in our study belong to three genera within the family Cyprididae: *Cypris* Müller, 1776, *Eucypris* Vávra, 1891, and *Heterocypris* Claus, 1893. *Cypris*, recognized as the oldest ostracod genera established under the Linnaean system, encompasses large‐bodied species (Mesquita‐Joanes et al. 2020). The biogeographical distribution of *Cypris* shows dynamism, with its highest species richness observed in Africa and Asia, and a notable presence in Europe (Meisch 2000; Mesquita‐Joanes et al. 2020). Previous investigations have documented 16 valid species within the genus *Cypris* including *C. pubera* found in this study. The genus *Eucypris* demonstrates extensive diversity worldwide, predominantly concentrated in the Palearctic Region (Meisch, Scharf, et al. 2019; Meisch, Smith, and Martens 2019; Batmaz et al. 2024). Turkey emerges as a hotspot for *Eucypris* diversity, serving as a crucial link between Europe and Asia (Külköylüoğlu et al. 2015; Batmaz et al. 2024). Haplotype network analysis of *E. virens* indicates a likely European origin for communities observed in Western Australia (Koenders et al. 2012). In our haplotype network, a prominent haplotype shared among Australia, China, and Europe exhibits a star‐shaped structure, indicative of its ancient lineage. The genus *Heterocypris* demonstrates a notable global distribution, with approximately 70 known species distributed widely in the Palearctic Region (Martens et al. 2019; Meisch, Scharf, et al. 2019; Meisch, Smith, and Martens  2019; Savatenalinton 2020; Smith and Chang 2020). Phylogenetic tree analysis conducted in earlier studies delineated 10 distinct clades (Pieri et al. 2013), with our findings belonging to clades A and D (A and H in this study), and introducing a novel clade (haplotype ZLPa in our study).

### 4.2 Mitonuclear Inconsistency in the Family Cyprididae

The detection of mitonuclear discordance within the family Cyprididae was observed in all three valleys, highlighting a prevalent phenomenon among freshwater crustaceans. Previous research has shown that mitonuclear discordance is common in this group of organisms, as evidenced by studies on various species (Thielsch et al. 2017; Liu et al. 2018; Cornetti et al. 2019; Ni et al. 2019) and nonmarine ostracods (Schön et al. 2018; Kilikowska et al. 2024). Several mechanisms have been proposed to explain mitonuclear discordance, including incomplete lineage sorting, gene flow through hybridization and introgression, different transmission modes, varying mutation rates, and the influence of natural selection on nuclear genes (Toews and Brelsford 2012; Després 2019). For example, a study on rotifers showed that hybridization played a role in creating mitonuclear discordance (Papakostas et al. 2016). Hybridization events have also been documented both between and within species of the family Cyprididae (Turgeon and Hebert 1995; Butlin et al. 1998), potentially leading to discordance between mitochondrial and nuclear genomes. The presence of the bacterial endosymbiont Cardinium is widespread among nonmarine ostracods including the family Cyprididae (Çelen et al. 2019; Schön et al. 2019; Schön and Martens 2020). It is believed that Cardinium may induce selective sweeps within genetic lineages of Cyprididae, resulting in the loss of mitochondrial diversity and contributing to the observed discrepancy between mitochondrial and nuclear genomes (Kilikowska et al. 2024).

### 4.3 Species Distribution Across Unique Valley Habitats

The species composition varied significantly among the three distinct valleys under investigation, with each region hosting its own endemic species (new lineage II, *Heterocypris* sp., and *H. salina* ) unique to their respective localities. There was no overlap of species across all three valleys, highlighting the spatial differentiation in species distribution likely due to the diverse environmental conditions in each habitat. The valleys, situated to the northwest, northeast, and southeast of Namtso Lake, are traversed by distinct watercourses, each presenting a unique environmental mosaic with singular ecological attributes (Guan and Chen 1980). Previous research on plankton biodiversity in the Nagqu River in the Tibetan Plateau has shown a correlation between changes in runoff composition and plankton biodiversity (Weng et al. 2020). Altitude disparities among the study areas (4798, 4597, and 3756 m) likely influenced the variations in species composition. Studies on the impact of elevational gradients on grassland community composition and structure on the Tibetan Plateau have revealed shifts in species composition with increasing elevation, with distinct discontinuity points at specific elevations (Wang et al. 2022). Furthermore, large‐scale elevation gradients can influence the diversity of aquatic plants, with taxonomic, phylogenetic, and functional diversity declining as altitude increases (Zhou et al. 2022).

### 4.4 Genetic and Geographical Dynamics of Ostracod Communities

Our study uncovers varying correlation patterns between genetic differentiation (*F*_ST_) and geographical distance in different regions. Specifically, RVI shows a significant positive linear correlation, whereas RVIII demonstrates a significant negative correlation. However, no significant relationship is found across the entire range or in RVII. These findings align with Ma et al. (2020) on zooplankton *Daphnia*, where a significant positive correlation with geographic distance is noted within a large spatial range but becomes insignificant within a smaller spatial range. In RVIII, a notable deviation from typical patterns is observed, with a significant negative correlation between genetic and geographical distances. Unlike the enclosed water bodies of RVI and RVII, sampling points in RVIII (excluding CCH) were mainly located along the Yarlung Zangbo River and its tributaries, defining an open water environment. Given the limited mobility of ostracods, which are planktonic organisms in aquatic ecosystems, the layout of river systems can profoundly impact their dispersal and genetic connectivity (Martens et al. 2008; Rahman et al. 2022). Connectivity of the water system emerges as a critical factor, surpassing mere geographical distance. For example, a study on the golden mahseer in Himalayan rivers shows that longitudinal connectivity significantly influences genetic differentiation among populations, emphasizing the importance of habitat connectivity (Yadav et al. 2020; Sharma 2020). Similarly, research on red mangrove indicates that complex hydrological connectivity leads to fine‐scale genetic structures, with local water flow regimes playing a crucial role in genetic diversity and differentiation (Chable Iuit et al. 2020). The Yarlung Zangbo River, characterized by lower overall altitude, developed transportation networks, and frequent population movements, experiences a more pronounced impact of human activities compared to the preceding regions (Li et al. 2015; Zhang et al. 2018; Liu et al. 2020), contributing to the eutrophication of water bodies (Nan et al. 2018; Liu et al. 2022) and posing risks to the composition, distribution, and diversity of zooplankton including ostracods (Liu et al. 2020). Ostracods produce dormant eggs that can be inadvertently transported by humans (e.g., via shoes, vehicles) to other water bodies, facilitating their colonization. For instance, insects of the family Orthosidae exhibit a similar capacity for long‐distance dispersal through unnatural means, leading to a mismatch between genetic and geographical distances observed in these insects (Valls et al. 2016; Szwarc and Namiotko 2022).

In total, 25 unique haplotypes were detected in the three river valleys, with no shared haplotypes observed among all three valleys. Phylogenetic analysis based on COI revealed three distinct groups representing nine clades, including two cryptic lineages, each showing discernible geographical distribution patterns. Interestingly, no correlation was found between genetic distance and geographical separation, highlighting altitude as a significant determinant of species distribution. Species composition varied significantly among the three river valleys, with each valley harboring its own dominant and endemic species. This study of the family Cyprididae on the Tibetan Plateau provides insights into the impact of river valleys on the genetic diversity and species distribution patterns of aquatic species.

## Author Contributions

**Qing Hu:** formal analysis (equal). **Bingkuan Zhu:** conceptualization (equal). **Zhihang Ma:** formal analysis (equal). **Yini Yang:** investigation (equal). **Zhixiong Deng:** resources (equal). **Shaoqing Wen:** funding acquisition (equal). **Xiaolin Ma:** writing – original draft (lead).

## Conflicts of Interest

The authors declare no conflicts of interest.

## Supporting information


Figure S1.
Figure S2.
Figure S3.
Figure S4.
Figure S5.
Figure S6.

Table S1.
Table S2.
Table S3.
Table S4.
Table S5.
Table S6.
Table S7.

## Data Availability

Data used for analysis of this article is included in Supporting Information.
