# Supplementary material for: Influence of River Valleys on Genetic Diversity and Species Distribution Patterns of Cyprididae (Crustacea: Ostracoda) on the Tibetan Plateau
Source: Ecol Evol. 2025 Jul 17;15(7):e71759. doi: 10.1002/ece3.71759 (PMC12268233; doi:10.1002/ece3.71759)

Figure S1. Haplotype network of *Cypris pubera* based on COI gene sequences. Each circle represents a unique haplotype, with the size of the circle proportional to the number of individuals sharing that haplotype. Colors within the circles indicate the geographical origin of each haplotype, as detailed in the legend.


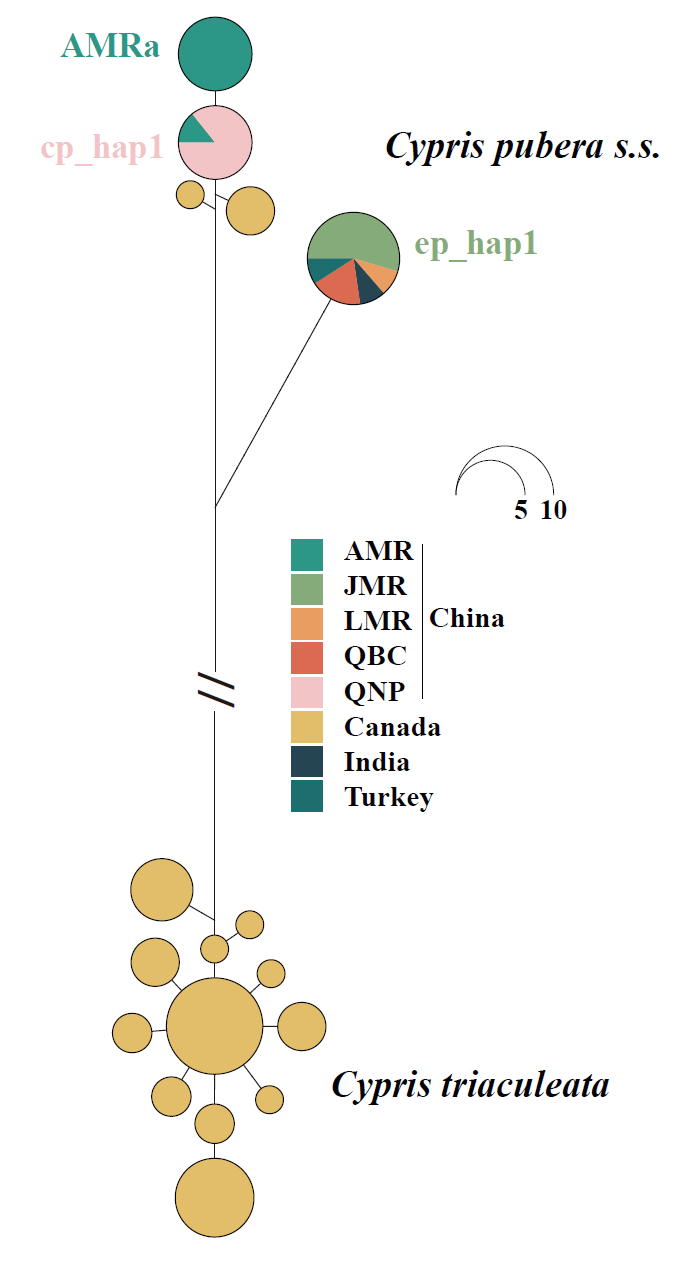


Figure S2. Haplotype network of *Eucypris virens* based on COI gene. Each circle represents a unique haplotype, with the size of the circle proportional to the number of individuals sharing that haplotype. Colors within the circles indicate the geographical origin of each haplotype, as detailed in the legend.


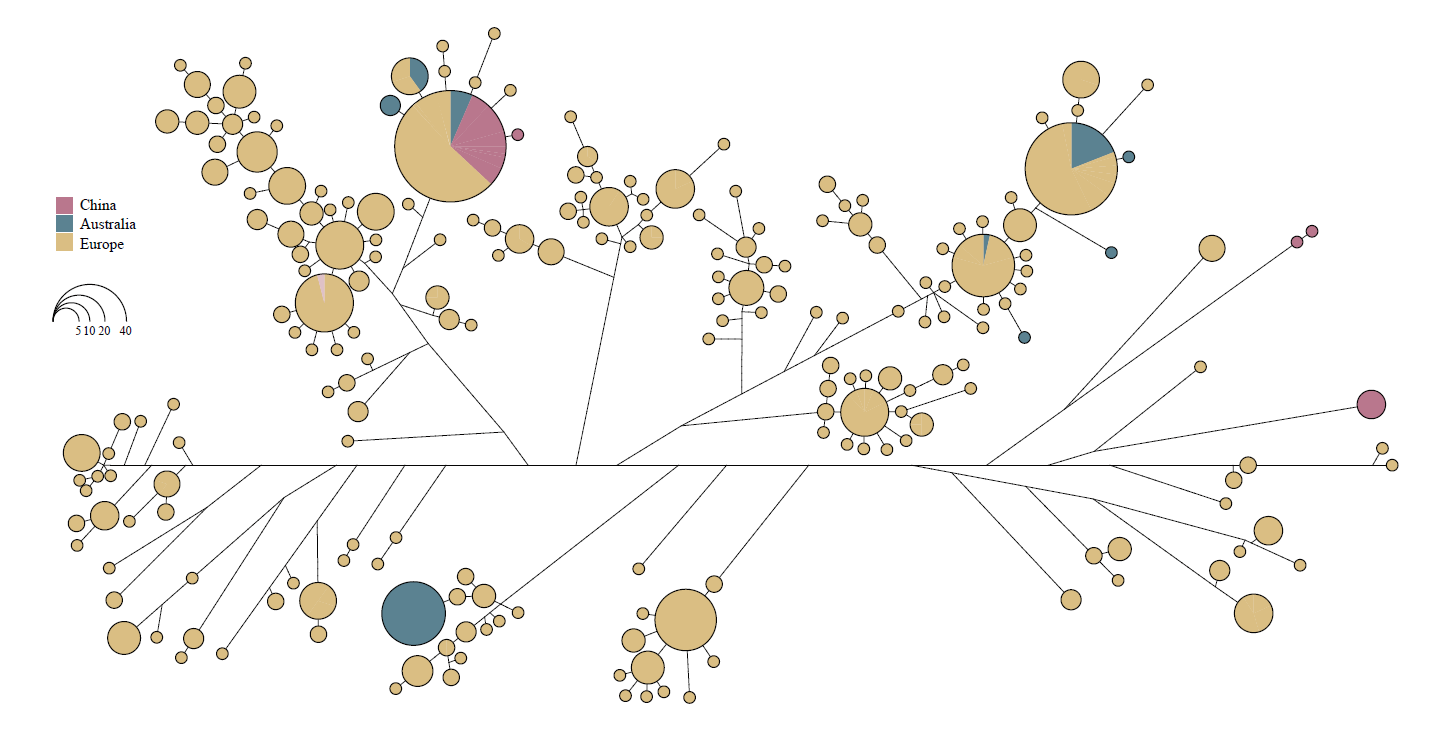


Figure S3. Haplotype network of *Heterocypris incongruens* based on COI gene. Each circle represents a unique haplotype, with the size of the circle proportional to the number of individuals sharing that haplotype. Colors within the circles indicate the geographical origin of each haplotype, as detailed in the legend.


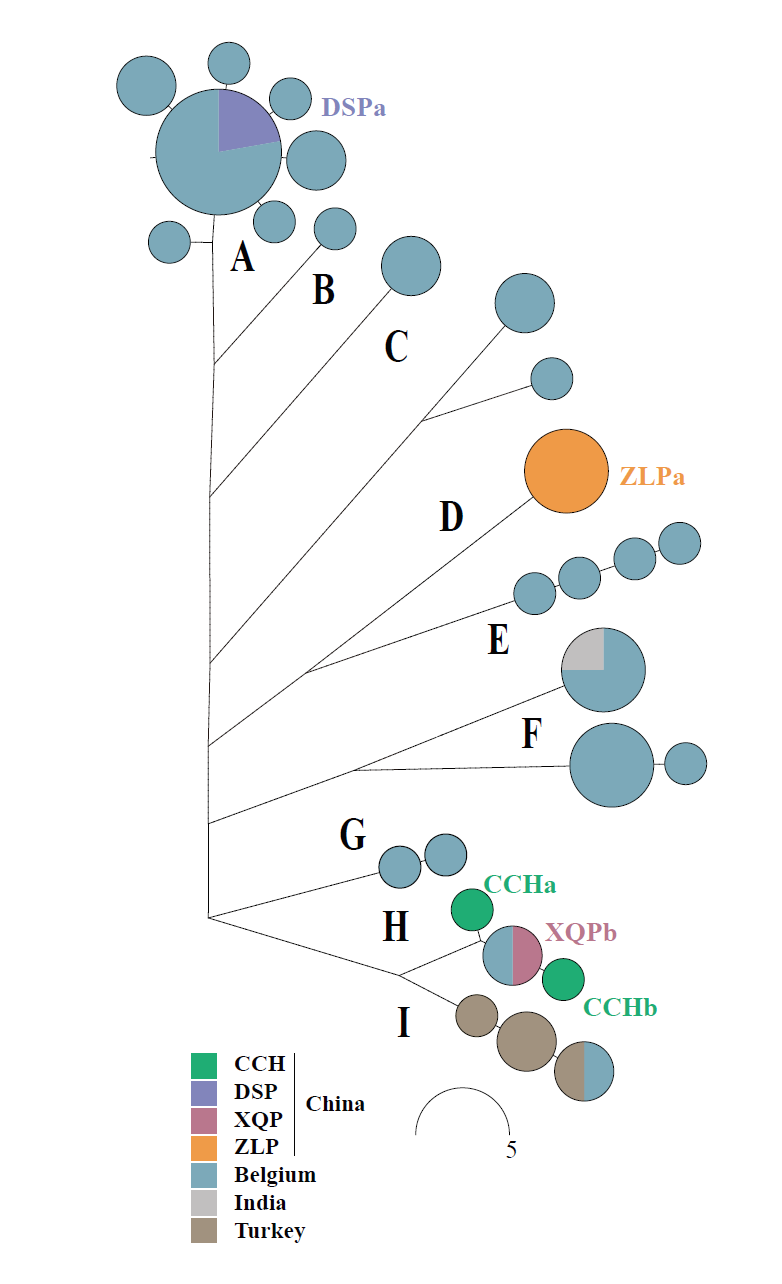


Figure S4. Phylogenetic trees based on COI (left) and 18S + 28S (right), showing mitochondrial-nuclear discordance in eight out of 15 communities (excluding three communities with insufficient data).


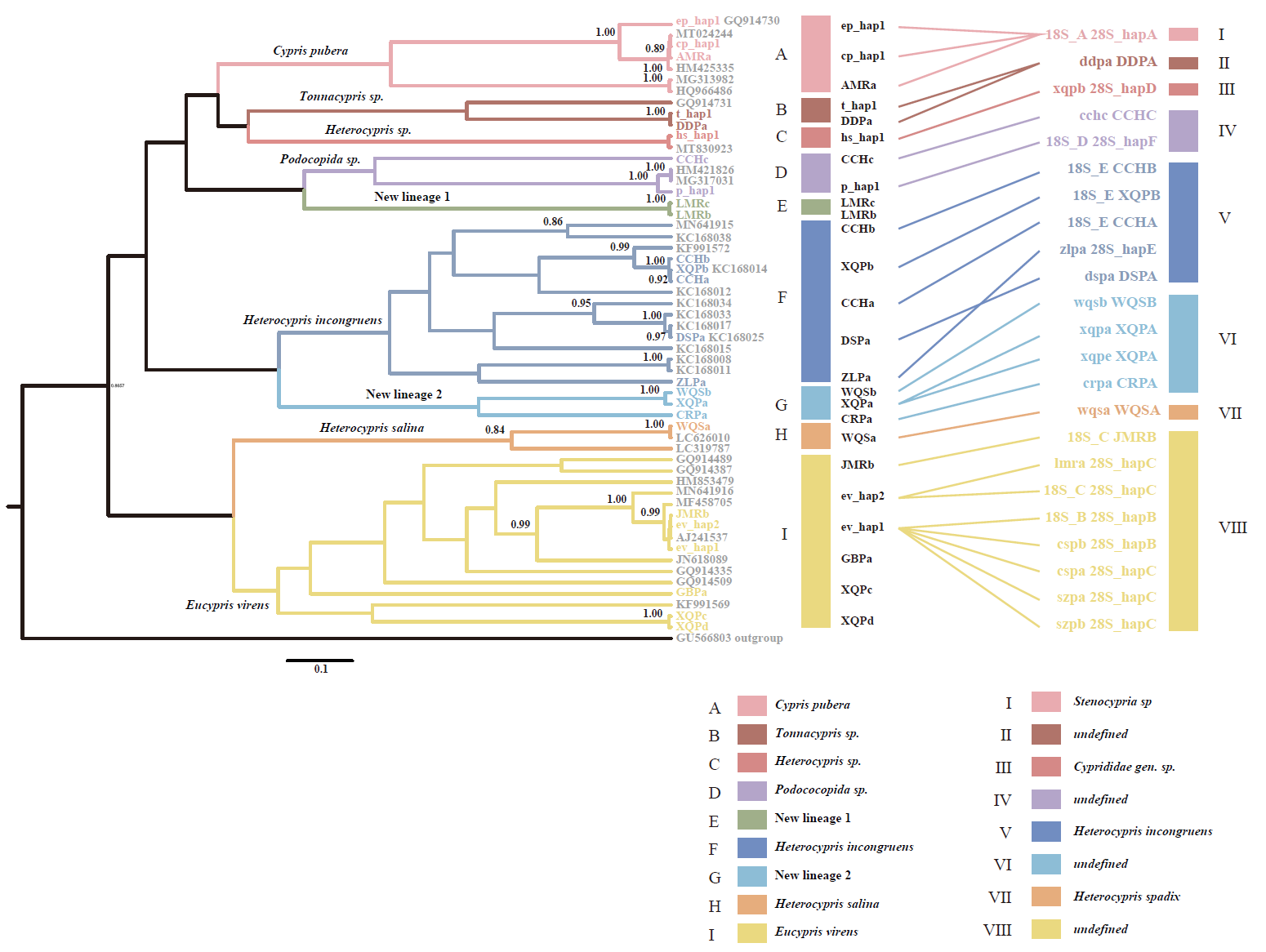


Figure S5. Phylogenetic trees based on 28S rRNA (A) and 18S rRNA (B) gene sequences, showing the relationships among different ostracod species. Each branch represents a different species or haplotype, with branch lengths indicating genetic distance. The colors and labels correspond to different species and geographic locations as indicated in the legend. The numbers on the branches represent bootstrap values, indicating the confidence level of each branch point. The pie charts next to certain branches illustrate the distribution of haplotypes across different regions.


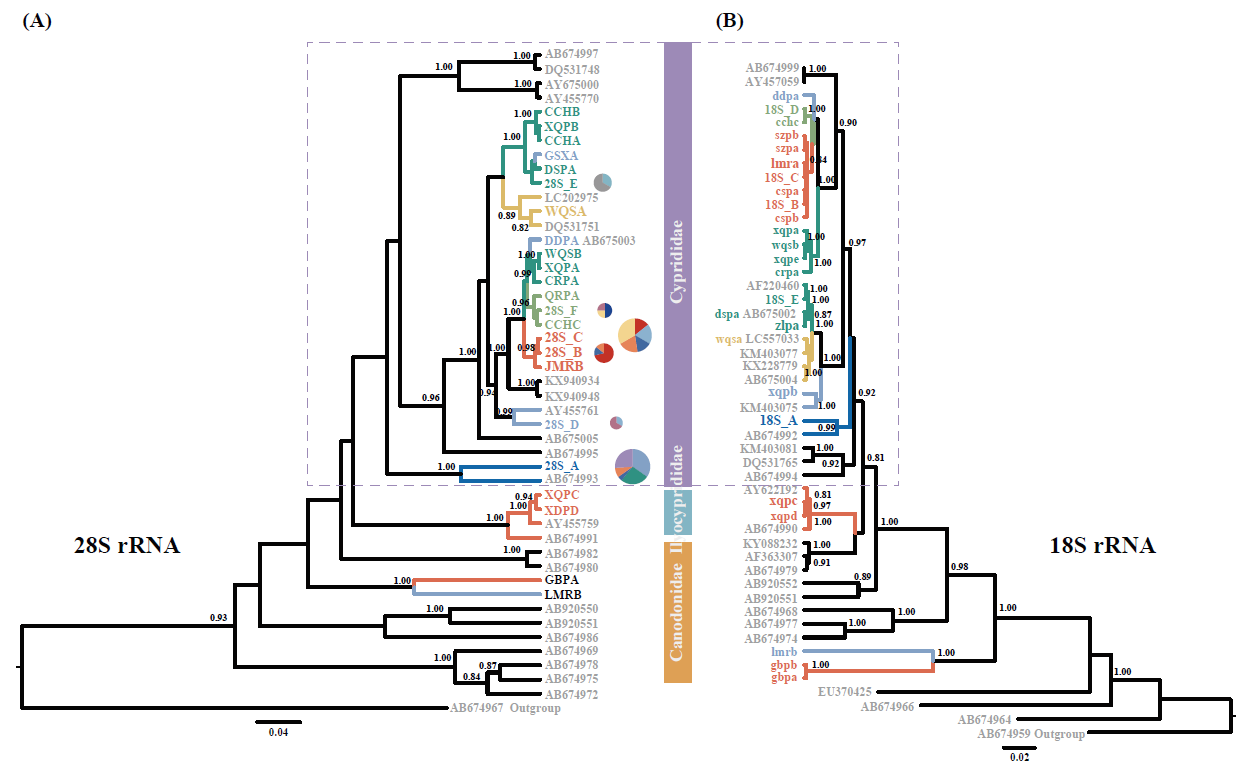


Figure S6. Principal Component Analysis (PCA) plot of the family Cyprididae (Crustacea: Ostracoda) from Tibetan Plateau, showing the relationship between genetic variation and geographical variables.


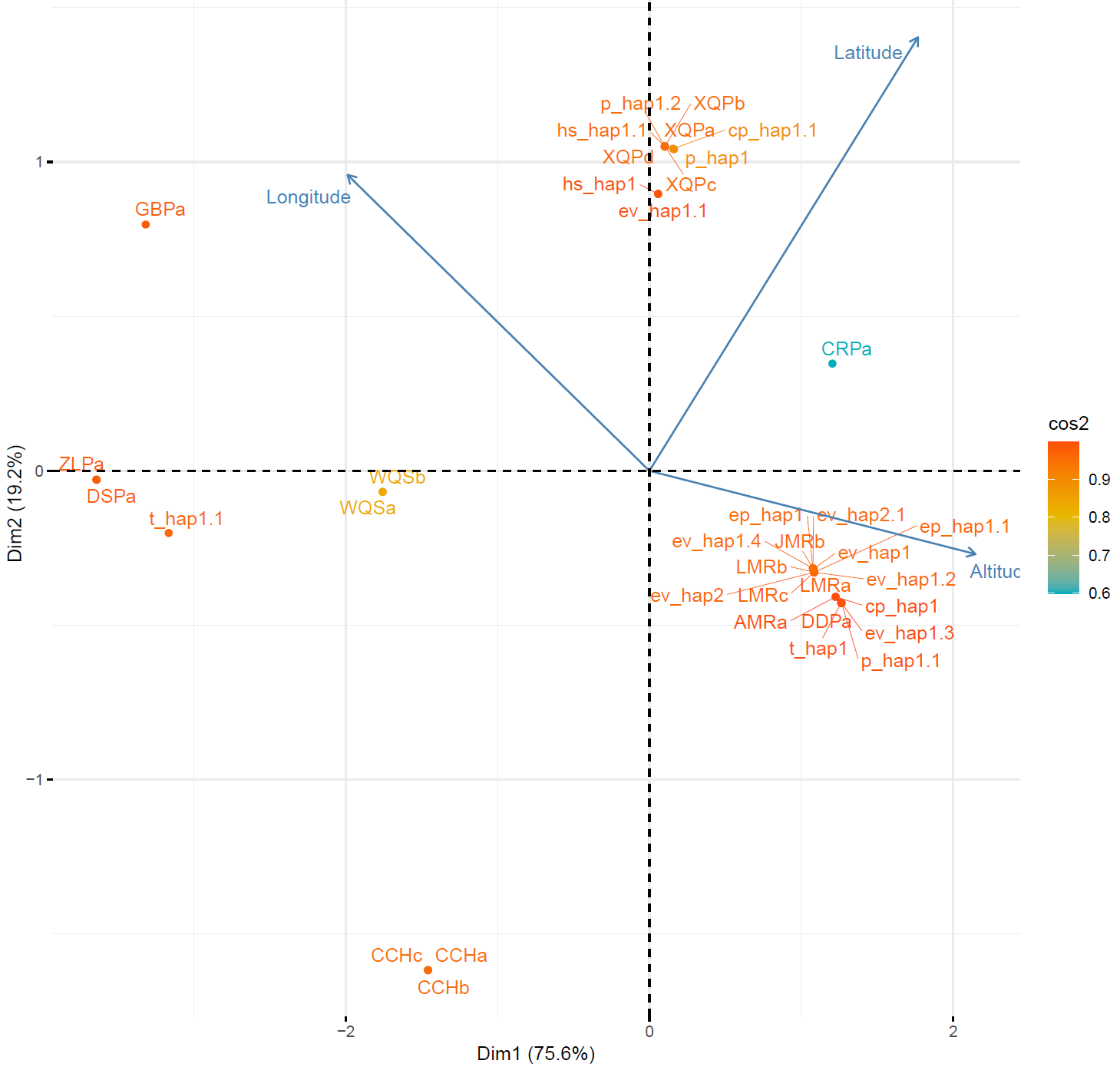

Supplement: Supplementary file 1 — Figure S1. Figure S2. Figure S3. Figure S4. Figure S5. Figure S6. [file ECE3-15-e71759-s002.docx]
